# Kinetic profile and mucosal capsular polysaccharide IgG responses to serotype 3 pneumococcus in the adult oropharynx following an experimental human challenge

**DOI:** 10.3389/fmicb.2026.1830972

**Published:** 2026-08-10

**Authors:** Ryan E. Robinson, Tinashe K. Nyazika, Ashleigh Howard, Sherin Pojar, Patricia Gonzalez-Dias, Elena Mitsi, James Court, Lisa Hitchins, Madlen Farrar, Konstantinos Liatsikos, Angela Hyder-Wright, Carla Solórzano, Britta C. Urban, Hassan Burhan, Elizabeth Begier, Christian Theilacker, Annaliesa S. Anderson, Bradford D. Gessner, Andrea M. Collins, Elissavet Nikolaou, Daniela M. Ferreira

**Affiliations:** 1 Department of Clinical Sciences, Liverpool School of Tropical Medicine, Liverpool, United Kingdom; 2 Respiratory Research Group, Liverpool University Hospitals Foundation Trust, Liverpool, United Kingdom; 3 Oxford Vaccine Group, NIHR Oxford Biomedical Research Centre, University of Oxford, Oxford, United Kingdom; 4 Pfizer Vaccines, Pfizer Inc., Collegeville, PA, United States; 5 Vaccines Research and Development, Pfizer Inc., Pearl River, NY, United States; 6 Infection, Immunity and Global Health, Murdoch Children's Research Institute, Parkville, VIC, Australia; 7 Department of Microbiology and Immunology, The University of Melbourne at the Peter Doherty Institute for Infection and Immunity, Parkville, VIC, Australia

**Keywords:** *Streptococcus pneumoniae*, serotype 3, carriage, nasopharynx, oropharynx, adults

## Abstract

**Background:**

*Streptococcus pneumoniae* serotype 3 (Spn3) is still a major cause of disease despite the implementation of the 13-valent pneumococcal conjugate vaccine. We previously reported on the development of an experimental human colonisation Spn3 model and showed that Spn3 was associated with frequent pharyngeal symptoms. Utilising this model, we compared the microbiological colonisation dynamics of Spn3 in the oropharynx and nasopharynx, along with associated symptoms and polysaccharide antibody responses.

**Methods:**

A total of 96 healthy adults (aged 18–50 years) were nasally inoculated with different dosages and strains of Spn3. Pneumococcal carriage frequency and density post-challenge were determined using culture and multiplex qPCR from nasal wash and saliva samples collected on days 2, 7, and 14 post-challenge. Spn3 density was compared with historical Spn6B data at day 2 post-challenge. IgG antibodies recognising the Spn3 capsular polysaccharide were measured at baseline and day 14 post-challenge in both niches.

**Results:**

Spn3 colonisation frequency was found to be similar in the nasopharynx and oropharynx on any day post-challenge, independently of challenge dose or strain tested. In saliva, Spn3 density was greatest at a challenge dose of 80,000 CFU/naris compared to the lowest or highest exposure doses. Of note, on day 2 post-challenge, Spn3 density was significantly higher than that observed following the Spn6B challenge at the same dose (*p* = 0.00090). No significant difference in Spn3 density was observed for challenge strain or symptomology on any day post-challenge. Spn3 polysaccharide-specific salivary IgG levels increased from baseline to day 14 in colonised participants (*p* = 0.0051) with a median IgG fold change of 1.458 [IQR 0.6131–4.281], but no increase in IgG levels occurred in uncolonized participants (*p* = 0.19). Participants colonised with the Spn3 clade Iα but not clade II had significant increases in polysaccharide-specific IgG from baseline (*p* = 0.0081).

**Conclusion:**

Given equivalent exposure doses, Spn3 colonised the oropharynx at higher densities than Spn6B. These data could help explain continued Spn3 population transmission despite widespread use of a Spn3-containing pneumococcal conjugate vaccine in the UK paediatric immunization programme. Within Spn3, clade Iα but not clade II increased mucosal IgG response following colonisation, which could help explain increasing transmission of clade II in the UK and higher transmission of this clade in mouse models.

## Introduction

*Streptococcus pneumoniae* (pneumococcus) commonly colonises the mucosal surfaces of the upper respiratory tract (URT) (Bogaert et al., 2004) without causing symptoms, a state referred to as asymptomatic colonisation (Weiser et al., 2018) or carriage. Pneumococcal carriage is detectable in the nasopharynx of approximately 40–90% of children (Bomar et al., 2018; Murad et al., 2019) and 10–25% of adults (Goldblatt et al., 2005) at any given time. In addition to serving as a reservoir for community transmission, URT colonisation may also act as an immunising event (Ferreira et al., 2013). Under certain conditions, pneumococcal spread can result in mucosal diseases such as otitis media, sinusitis, conjunctivitis, and non-bacteraemic pneumonia, or progress to invasive diseases including bacteraemic pneumonia, septicaemia, and meningitis (Weiser et al., 2018).

In children, pneumococcal colonisation occurs predominantly in the nasopharynx (Bomar et al., 2018), whereas in adults, colonisation can occur in both the nasopharynx and oropharynx (Levine et al., 2012; Lieberman et al., 2006; Watt et al., 2004). Studies assessing pneumococcal acquisition in adults using oral and nasal sampling have reported variable detection rates (Watt et al., 2004; Gladstone et al., 2012; Arguedas et al., 2020). We previously demonstrated superior detection of experimental pneumococcal serotype 6B (Spn6B) colonisation using nasal wash samples compared with oropharyngeal swabs (Nikolaou et al., 2021). Carriage dynamics also vary between pneumococcal serotypes (Chaguza et al., 2021), and even strains within the same serotype can differ in epithelial adherence capacity in vitro (Cundell et al., 1995).

Pneumococcus serotype 3 (Spn3) is one of the most prevalent circulating SPN serotypes even in countries with mature infant 13-valent pneumococcal conjugate vaccine (PCV13) immunisation programmes (Adler et al., 2019) and remains a common cause of community-acquired pneumonia (CAP) (Pick et al., 2020). Surveillance studies have reported that the population-level incidence of invasive disease due to Spn3 has remained static despite dramatic reductions in other serotypes included in PCV13 (Løchen et al., 2020) and despite documented direct protection against Spn3 disease among vaccinated adults (Gessner et al., 2019; Warren and Weinberger, 2020) and children (Sings et al., 2019). One explanation for this constellation of results is that PCV13 protects against disease but insufficiently against carriage, and thus transmission continues relatively unabated; this, in turn, may be exacerbated by differences in Spn3 clades (Groves et al., 2019; Azarian et al., 2018; Sekulovic et al., 2024). Spn3 exhibits distinct colonisation biology characterised by extensive capsular production, immune evasion, capsule shedding, and relatively poor immunogenicity, which together may contribute to persistent carriage and ongoing disease burden despite vaccine inclusion (Luck et al., 2020).

The Experimental Human Pneumococcal Challenge (EHPC) model, in which participants are experimentally inoculated nasally with pneumococcus and then monitored for nasopharyngeal colonisation (Ferreira et al., 2013), has been expanded to include Spn3 (Robinson et al., 2022). Rates of colonisation and levels of bacterial densities recovered from the nasopharynx in this Spn3 model were comparable to previously reported serotype 6B experimental colonisation studies (Robinson et al., 2022). However, participants frequently reported mild symptoms post-challenge, particularly sore throat (Robinson et al., 2022), which had not previously been observed with SPN6B^6^. Given the frequency of pharyngeal symptoms observed during Spn3 colonisation, we hypothesised that Spn3 could have a preferential niche for colonisation in the oropharynx and therefore be more frequently identifiable and at higher density in saliva compared with Spn6B. Here we evaluated Spn3 colonisation kinetics (prevalence at days 2, 7, and 14) and density, associations with symptoms and mucosal anti-capsular IgG responses, and differences between Spn3 clades and compared them to historic data on Spn6B obtained from the EHPC.

## Methods

### Study design and subjects

**Figure 1:**
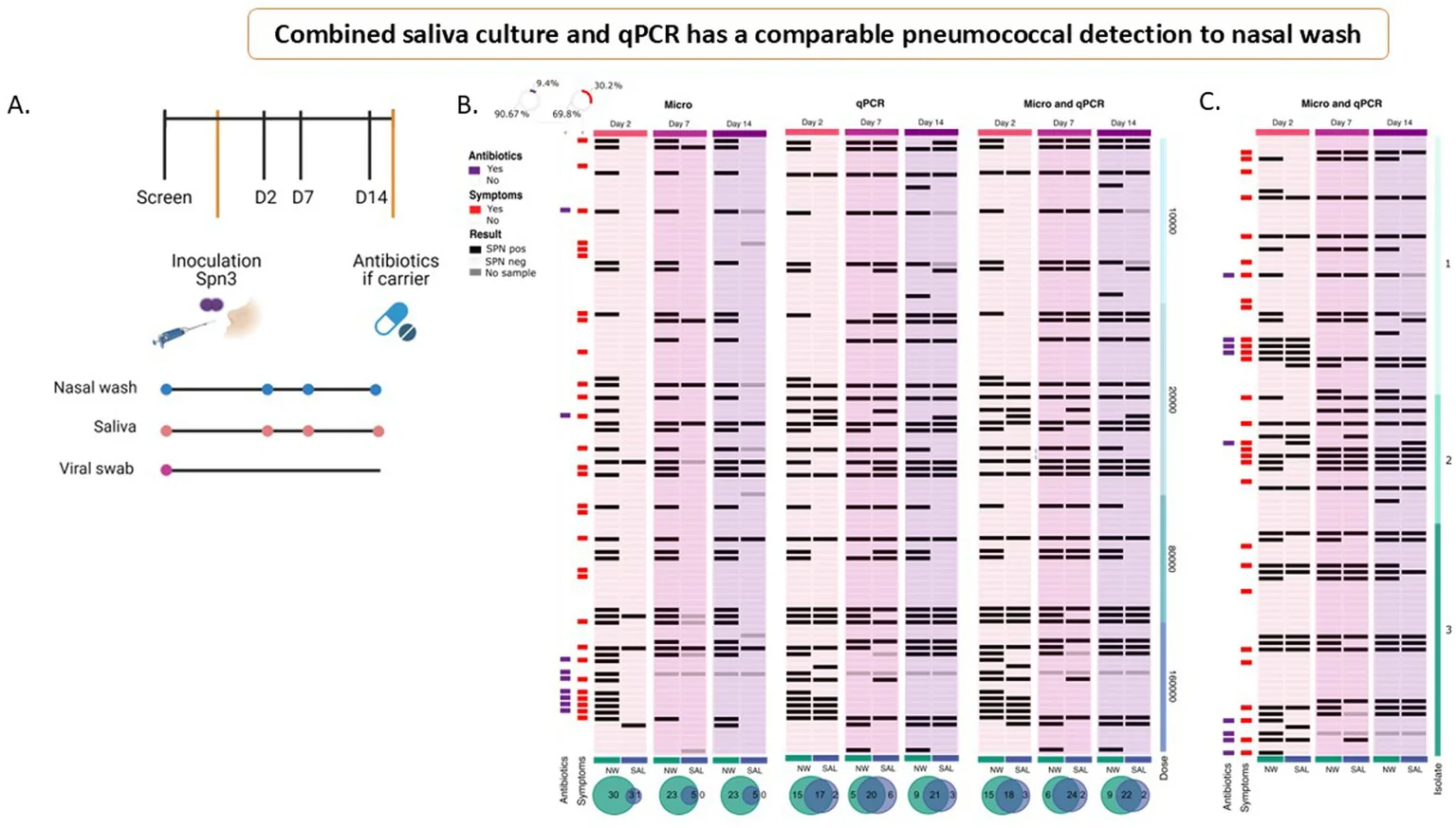
Experimental Spn3 colonisation in nasal wash and saliva using culture and molecular methods. **(A)** A schematic diagram of sampling timepoints. **(B)** Acquisition of SPN3 colonisation at days 2 [in rose], 7 [in pink], and 14 [in purple] for all participants (*n* = 96). Saliva and NW culture and qPCR results are shown and combined. SPN3-positive participants are in black, negative in light grey, and missing samples in dark grey. Symptomatic participants are identified in red with a donut graph summary. Venn diagram of a sample agreement between NW and saliva is shown at the bottom of each row. **(C)** Acquisition stratified by challenge strain using microbiology and qPCR methods combined.

The study design and methodology for the Spn3 challenge model have been previously described (Robinson et al., 2022). In brief, healthy participants aged 18–50 were recruited between November 2019 and March 2021; the study was paused between March 2020 and January 2021 due to the COVID-19 pandemic. Eligible participants were nasally inoculated, with 100 μL per nare of either a penicillin sensitive live Clonal Complex 180 (CC180) Spn3 strain PF ESP306 (clade Iα), PF ESP505 (clade II) or non-CC180 PF ESP231 (no identifiable clade, referred to as ‘no clade’) (Antimicrobial Testing Leadership and Surveillance database; Pfizer, National Center for Biotechnology Information BioSample accessions SAMN27406322, SAMN27406323, SAMN27406324). The inoculum dose was escalated based on the safety profile and achieved colonisation rates. Inoculum doses of 10,000 colony-forming units (CFU) per nostril, 20,000 CFU, 80,000 CFU, and 160,000 CFU were used in 10 participants per group. Follow-up and sampling occurred on days 2, 7, and 14 post-challenge (Figure 1A). Participants who became experimentally colonised received antibiotics at their last study visit (day 14). All participants underwent safety screening. The study was prospectively registered on the International Standard Randomised Controlled Trial Number database (ISRCTN11306486).

### Nasal wash and saliva sampling

NW and saliva samples were collected at each visit pre- and post-Spn3 challenge. Samples were collected in the following order: (1) saliva and (2) NW from all participants. To obtain NW samples, 10 mL of 0.9% sodium chloride solution (Algeos, Liverpool, UK) was introduced per nostril using a syringe and held in the participant’s nose for a few seconds before being expelled into a sterile container, as per previous EHPC studies (Ferreira et al., 2013). NW samples were cultured fresh on the day of collection to increase detection sensitivity for pneumococcus, and the remaining NW were frozen at −80 °C until use in molecular testing. For saliva samples, participants were asked to spit approximately 1 mL of saliva into a sterile container containing 1 mL of skimmed milk-tryptone-glucose-glycerine (STGG). Saliva samples were frozen at −80 °C for later use in both microbiological culture and molecular testing.

### Spn3 detection in NW and saliva samples by culture

On the day of collection, NW samples were centrifuged for 10 min at 2,719x*g*, with the supernatant collected and cultured, whilst the remainder of the pellets were resuspended in 800 μL of STGG. NW pellets were quantitatively cultured on Columbia blood agar supplemented with 5% horse blood (Oxoid/Thermo Scientific) and 80 μL of gentamicin 1 mg/mL (Sigma-Aldrich). The plates were incubated at 37 °C and 5% CO_2_ overnight, and Spn3 colonies were confirmed as *S. pneumoniae* by their morphology, sensitivity to optochin disk, and solubility in bile salts. Serotyping was performed by latex agglutination (SSI Diagnostica, UK) to confirm the pneumococcal serogroup. For cultured saliva, frozen saliva samples were thawed for 20 min at room temperature, vigorously vortexed for 20 s, then centrifuged and cultured as per the NW protocol. The remaining NW and saliva samples in STGG were frozen at −80 °C until DNA extraction.

### Spn3 DNA extraction of NW and saliva samples in STGG

Prior to DNA extraction, the samples were thawed at room temperature before being vigorously vortexed for 20 s. For NW, a 200 μL aliquot was used for DNA extraction. For saliva, the samples were first centrifuged at 2,719x*g* for 15 min. The supernatant was collected, and the pellet was resuspended in 400 μL of STGG and used for DNA extraction. NW and saliva aliquots were centrifuged at 20,238x*g* for 10 min, followed by the addition of 300 μL of lysis buffer with protease (LGC Genomics, UK), 100 μL of zirconium beads (diameter of 0.1 mm), and 300 μL of phenol pH 8.0. The samples were mechanically disrupted at 50 Hz for 3 min in a tissue homogenizer, followed by 3 min on ice. This cycle was repeated twice. The samples were then centrifuged for 10 min at 9,391x*g*, and the upper aqueous phase was transferred to a sterile 1.5 mL Eppendorf tube pre-filled with 600 μL of binding buffer and 10 μL magnetic beads. The samples were incubated in a mixing machine for 1 h at room temperature before being washed twice with 200 μL of wash buffers 1 and 2. Magnetic beads were dried at 55 °C for 10 min and eluted in 63 μL of elution buffer. DNA was stored at −20 °C until use in quantitative polymerase chain reaction (qPCR).

### Quantification of Spn3 DNA by multiplex-qPCR in NW and saliva pellets

Multiplex-qPCR targeting the *lytA* (Carvalho et al., 2007) and *cps-3* genes (Azzari et al., 2010) was utilised and extracted DNA samples were run on a QuantStudio 5 machine (Thermo Fisher Scientific, UK). TIGR4 DNA extracted using the QIAamp DNA mini kit (Qiagen, UK) and serially diluted 1:10 from 4.14 × 10^6^ copies in 2.5 μL was used for the standard curve. A negative DNA extraction control (parallel extraction from sample buffer only), a qPCR negative control (master mix only), and a qPCR positive control (pneumococcal Spn3 strain) were amplified in every run. The samples were run in duplicate and considered positive only if both genes had a C_T_ < 40 cycles. Manual thresholds were set for *lytA* at 0.132 and for *cps3* at 0.04.

To avoid false-positive results from non-Spn3-like strains, *lytA* and *cps-3*-positive samples also underwent a singleplex qPCR targeting the *pia*B (Trzciński et al., 2013) gene. For the latter, the samples were run in triplicate and considered *pia*B positive if at least two of the three replicates had a C_T_ < 40 cycles. A relative threshold was set to default to normalise values within plates. Only samples positive for all three genes (*lyt*A, *cps*-3, and p*ia*B) were considered Spn3-positive and used in this analysis.

### Detection of Spn3 anti-pneumococcal capsular polysaccharide antibodies in NW and saliva samples

The WHO-standardized enzyme-linked immunosorbent assay (ELISA) was used (Goldblatt et al., 2011). ELISA plates were coated with 5 μg/mL of Spn3-purified capsular polysaccharides (CPS) (Statens Serum Institute) and stored overnight at 4 °C. NW and saliva samples were diluted in an absorption buffer containing 10 μg/mL CWPS multi (Statens Serum Institute). Following adsorption, samples were transferred to pre-coated plates and incubated for 2 h at room temperature. Antigen-specific antibodies were detected using anti-human biotinylated IgG, HRP-streptavidin, and tetramethylbenzidine (TMB) substrate-based platform. The reaction was stopped with 2 N H_2_SO_4_, and the optical density was measured at 405 nm using a FLUOstar Omega plate reader (BMG Labtech). All samples were run in duplicate.

### Identification of participant symptoms post-challenge

Participants were questioned for the presence and onset of pre-specified symptoms (Robinson et al., 2022) (any respiratory symptoms, headache, earache, sore throat, fever >38 °C) at each study visit and, if reported, underwent review by a clinician and were followed up until resolution. Symptomatic participants were defined as those self-reporting symptoms during the study follow-up period. Antibiotics (amoxicillin) were initiated if symptoms were escalating in severity or significantly troublesome as judged by a clinician.

### Statistical methods and analysis

Analyses were performed in GraphPad Prism (version 9.20) and R (version 4.0.4). Summary statistics were reported using descriptive statistics. Detection of carriage was compared using contingency tables, and the NW and saliva association was assessed using Fisher’s exact test. Cohen’s Kappa was used to measure the agreement between sample methods. For parametric data, paired and unpaired t-tests were used, and for non-parametric data, the Wilcoxon and Mann–Whitney tests were used, where appropriate. For Wilcoxon’s tests, no additional multiple-testing correction was applied beyond the default multiple-comparison adjustment implemented in GraphPad Prism. Multiplicity-adjusted *p*-values were reported for all pairwise comparisons included in each analysis. Correlation (r) was assessed using Spearman’s rank for non-parametric data and Pearson’s correlation coefficient for parametric data. Data were log_10_ transformed where appropriate. Differences were considered significant at a *p*-value of <0.05, and multiple testing correction was used where appropriate.

## Results

### Experimental Spn3 was detected at comparable rates in both saliva and nasal wash samples post-challenge using culture and qPCR methods in combination

A total of 96 healthy participants were recruited as previously described (Robinson et al., 2022). Participant journey and samples collected are shown (Figure 1A). All Spn3 challenge inoculum doses had 10 participants per dose group (except for the clade II strain at 10,000 CFU/naris, where *n* = 6). Paired NW (*n* = 286) and saliva (*n* = 283) samples were obtained at days 2 (NW *n* = 96 vs. saliva *n* = 96), 7 (NW *n* = 95 vs. saliva *n* = 94), and 14 (NW *n* = 95 vs. saliva *n* = 93) following the experimental Spn3 challenge. Using culture, qPCR, or both in combination, we identified participants with pneumococcal colonisation at each timepoint post-challenge using NW and saliva samples (Figure 1B), and for all challenge strains and dose-ranging groups.

Considering culture and qPCR data combined, overall, 32.9% (94/286) of NW samples were positive for experimental Spn3, whereas 25.1% (71/283) of saliva samples were positive. Spn3 was detected at comparable proportions in saliva and NW with no significant differences between the two sampling niches on any day post-challenge (day 2, *p* = 0.076; day 7, *p* = 0.633; day 14, *p* = 0.338) (Figure 1B). The challenge dose did not affect the rates of Spn3 detection between NW and saliva (Figure 1B). Similarly, no difference was seen when stratified by challenge strain at all time points (Figure 1C).

We also assessed the level of agreement between the paired sample types for pneumococcal detection. Moderate agreement was observed at day 2 (Kappa = 0.545, 95% CI 0.367–0.723), and substantial agreement on days 7 (Kappa = 0.796, 95% CI 0.662–0.930), 14 (Kappa = 0.717, 95% CI 0.563–0.871), and overall (Kappa = 0.805, 95% CI = 0.685–0.926). Fewer positive samples were identified at days 7 and 14, possibly due to the requirement for antibiotics at day 2 in seven colonised participants in the 160,000 CFU/naris dose group to treat URI symptoms.

When culture was used alone, experimental Spn3 was detected significantly more frequently in NW than concurrent saliva sampling on any day post-challenge (day 2 saliva = 4/96 vs. NW = 33/96, *P* = <0.0001; day 7 saliva = 5/90 vs. NW = 28/95, *P* = <0.0001; day 14 saliva = 5/89 vs. NW = 28/95, *P* = <0.0001). The use of qPCR methods improved the detection of Spn3 in saliva at all timepoints [day 2 qPCR only = 19/96 (19.8%) vs. culture only = 4/96 (4.1%), day 7 qPCR only = 26/94 (27.6%) vs. culture only = 5/90 (5.6%), day 14 qPCR only = 25/93 (26.9%) vs. culture only = 5/89 (5.6%)].

### Densities of Spn3 in saliva are higher than previously observed following SPN6B colonisation, but did not differ with strain or symptomology

**Figure 2:**
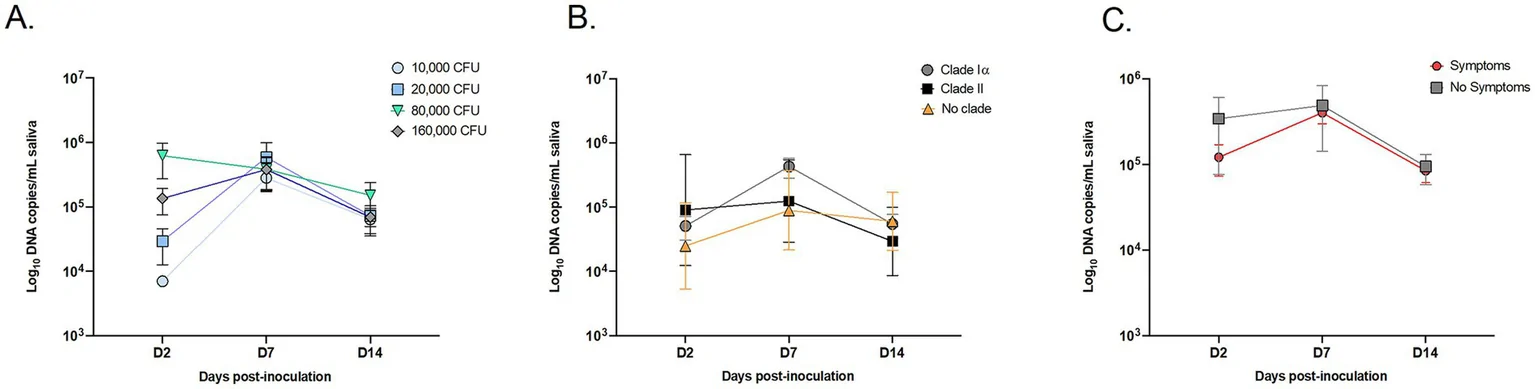
Spn3 density in saliva post-challenge at days 2, 7, and 14. **(A)** Mean bacterial density in saliva with standard error margins, stratified by dose group. Reported DNA copies/mL are log-transformed. Density calculations utilised the *piaB* gene. **(B)** Density in saliva samples stratified by challenge strain. Reported DNA copies/mL are log-transformed. Density calculations utilised the *piaB* gene. **(C)** Density in saliva samples from those with symptoms (in red) and those without (in grey). Reported DNA copies/mL are log-transformed. Density calculations utilised the *piaB* gene.

To ascertain if colonisation density in saliva differed between the challenge doses, we compared Spn3 density in saliva at each time point. Densities are presented and analysed after log_10_ transformation. At day 2 post-challenge, the recovered bacterial densities were highest in those inoculated with 80,000 CFU/naris [mean log_10_ density; 5.53 (95% CI 4.55–6.52)] when compared to the 20,000 CFU/naris [mean log_10_ density; 4.01 (95% CI 3.45–4.56)] and 160,000 CFU/naris [mean log_10_ density; 4.89 (95% CI 4.43–5.35), *p* = 0.005; Figure 2A]. At 10,000 CFU/naris, only one individual was colonised at day 2, so we excluded them from the analysis. No differences in bacterial densities by dose were observed on days 7 (all *p >* 0.05) or 14 (all *p* > 0.05).

We also assessed the recovered Spn3 bacterial density by challenge strain. No significant differences were observed post-challenge in bacterial densities (all *p* > 0.05; Figure 2B). Similarly, no significant differences in recovered bacterial densities were observed between symptomatic and asymptomatic individuals following the challenge at all time points (day 2, *p* = 0.891; day 7, *p* = 0.169; day 14, *p* = 0.649) (Figure 2C).

**Figure 3:**
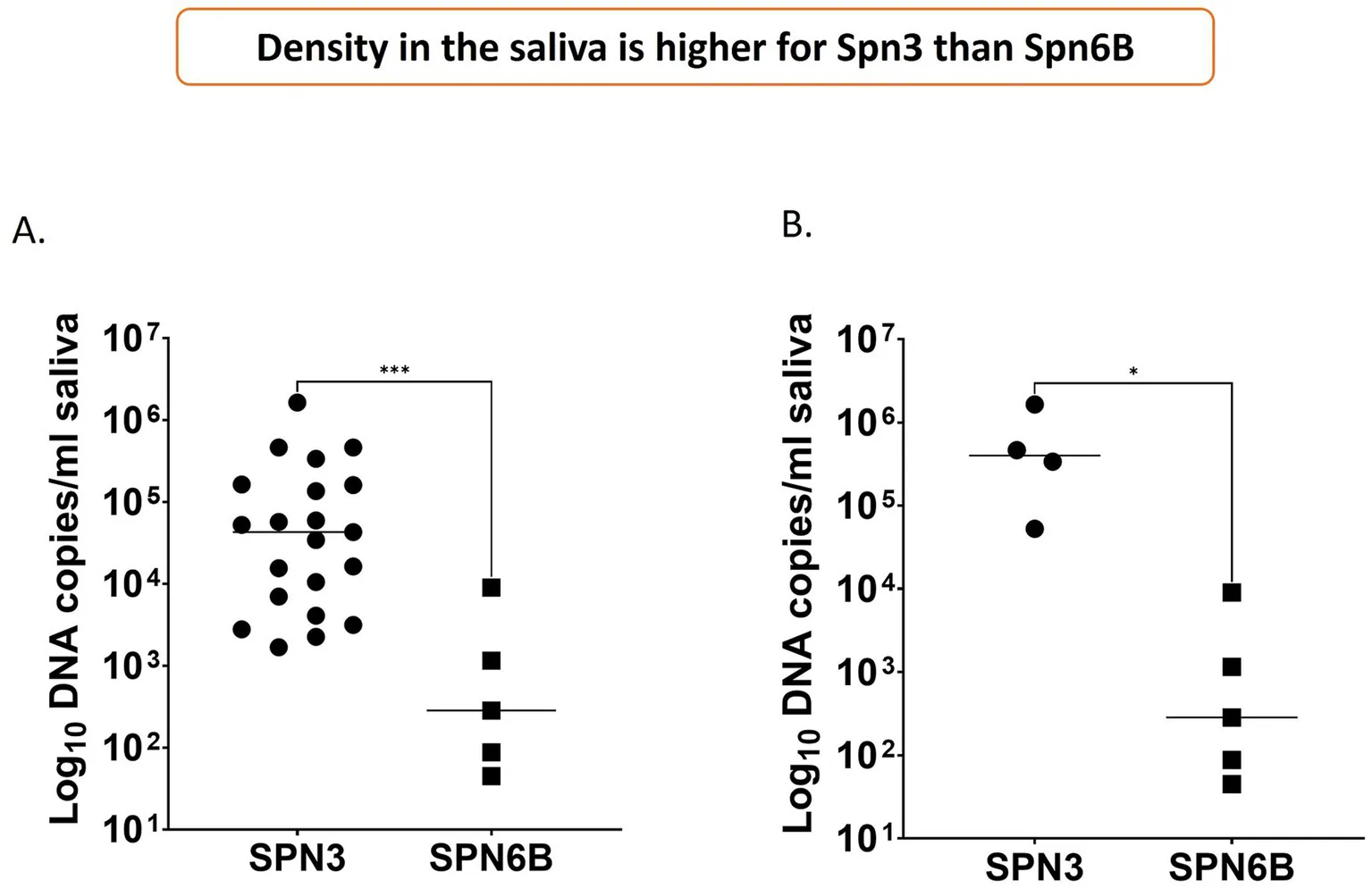
Comparison of density in saliva post-Spn3 and Spn6B challenge at day 2. **(A)** Density in saliva samples following Spn3 (dosing ranging from 10,000 to 160,000 CFU/naris) and Spn6B challenge (80,000 CFU/naris). Reported DNA copies/mL are log-transformed. Density calculations utilised the *PiaB* gene for Spn3 and the *6AB* gene for Spn6B. Comparisons were performed using the Mann–Whitney test. **(B)** Density in saliva samples following Spn3 (at 80,000 CFU/naris) and Spn6B challenge (80,000 CFU/naris). Density calculations utilised the *PiaB* gene for Spn3 and the *6AB* gene for Spn6B. Comparisons were performed using the Mann–Whitney test.

The density in saliva following the Spn3 challenge was compared to historical Spn6B challenge data^,^ (Adler et al., 2021; Rylance et al., 2019) on day 2 post-challenge and found to be significantly higher (*P* = <0.001; Figure 3A; Spn3 dosing ranging from 10,000 to 160,000 CFU/naris and Spn6B at 80,000 CFU/naris and B; both Spn3 and Spn6B at 80,000 CFU/naris).

### Spn3 colonisation elicits an increase in anti-CPS IgG levels in the oral mucosa

**Figure 4:**
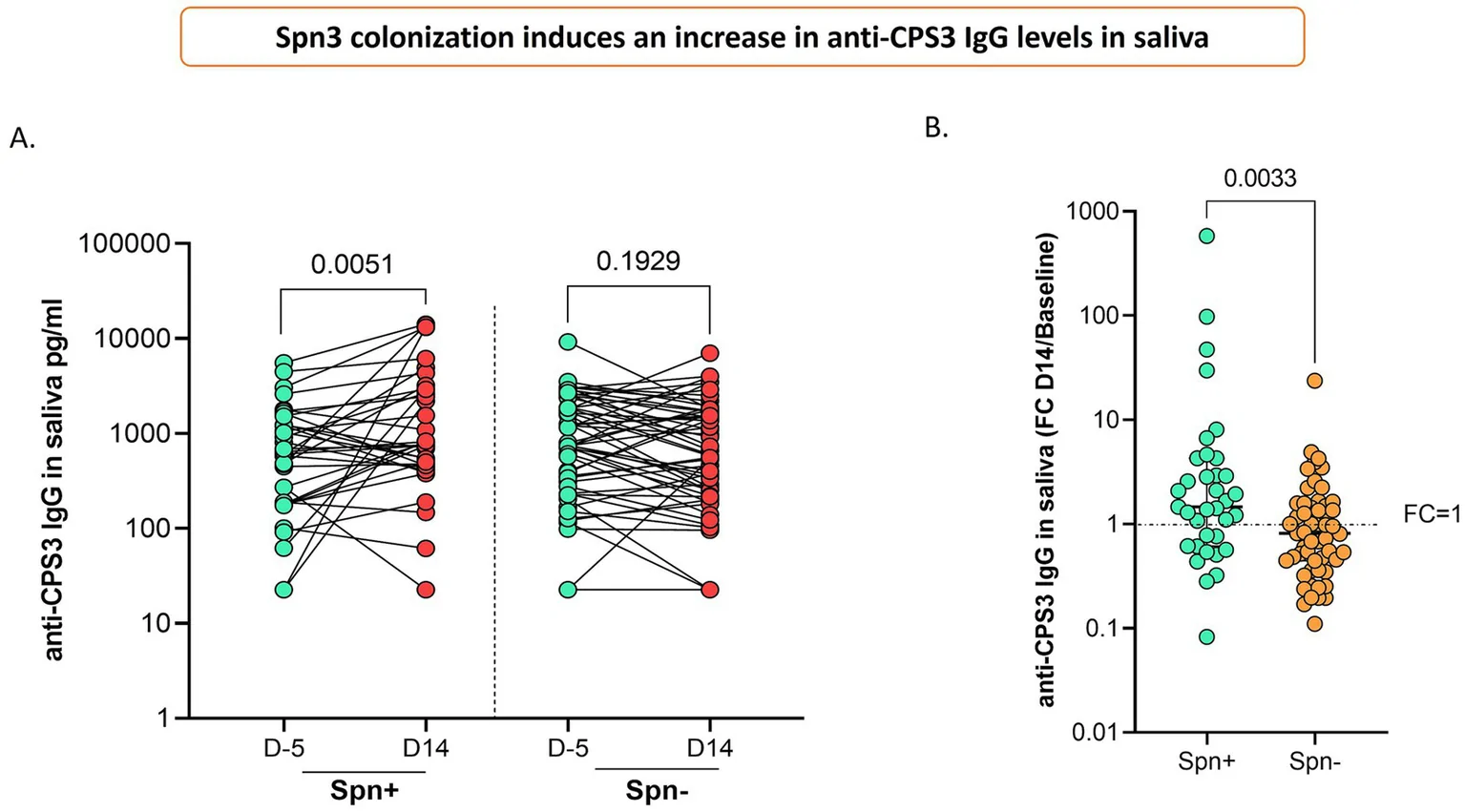
Anti-CPS3 IgG response detected in saliva for colonised and non-colonised participants. **(A)** Spn3 anti-capsular IgG in saliva at baseline (green) and day 14 (red) for colonised and non-colonised. Comparisons were performed using the Wilcoxon test. **(B)** Comparison of fold change in saliva anti-CPS3 IgG in colonised (green) and non-colonised participants (orange). Comparisons were performed using the Mann–Whitney test. Abbreviations: FC D14/baseline = fold change between day 14 post-challenge and baseline levels.

Paired NW and saliva samples collected at baseline (*n* = 86) and day 14 (*n* = 86). When colonised and non-colonised participants were compared, a significant increase in salivary IgG anti-CPS3 was observed by day 14 from baseline in the colonised group [geometric mean concentration (GMC) IgG at baseline = 537.4 pg./mL (GMC 95% CI 345.8–835.1) vs. GMC IgG at day 14 = 1,015 pg./mL (GMC 95% CI 619.9–1,663), *p* = 0.0051, Supplementary Table 1]. No significant IgG increase was observed in non-colonised participants at day 14 compared to baseline [GMC IgG at baseline = 633.8 pg./mL (GMC 95% CI 432.3–929.2) vs. GMC IgG at day 14 = 523.3 pg./mL (GMC 95% CI 352.4–777.2); *p* = 0.19; Figure 4A]. Fold change (FC) in IgG pre- and post-challenge, accounting for the varying baseline levels, also showed that colonised participants had a higher induction of IgG compared to non-colonised participants [colonised participants median IgG FC = 1.458 (IQR 0.613–4.28), non-colonised median IgG FC = 0.812 (IQR 0.442–1.488), *p* = 0.003; Figure 4B]. 13/38 participants colonised did not elicit an observable IgG response (a fold change >1 from baseline to day 14).

Spn3 saliva density did not correlate with saliva anti-CPS3 IgG (day 2 *r* = −0.392 *p* = 0.087, day 7 *r* = 0.261 *p* = 0.188, day 14 *r* = −0.052 *p* = 0.791, Supplementary Figures 1A–C).

### High dose of bacterial challenge induces an increased Spn3 IgG level response to colonisation

**Figure 5:**
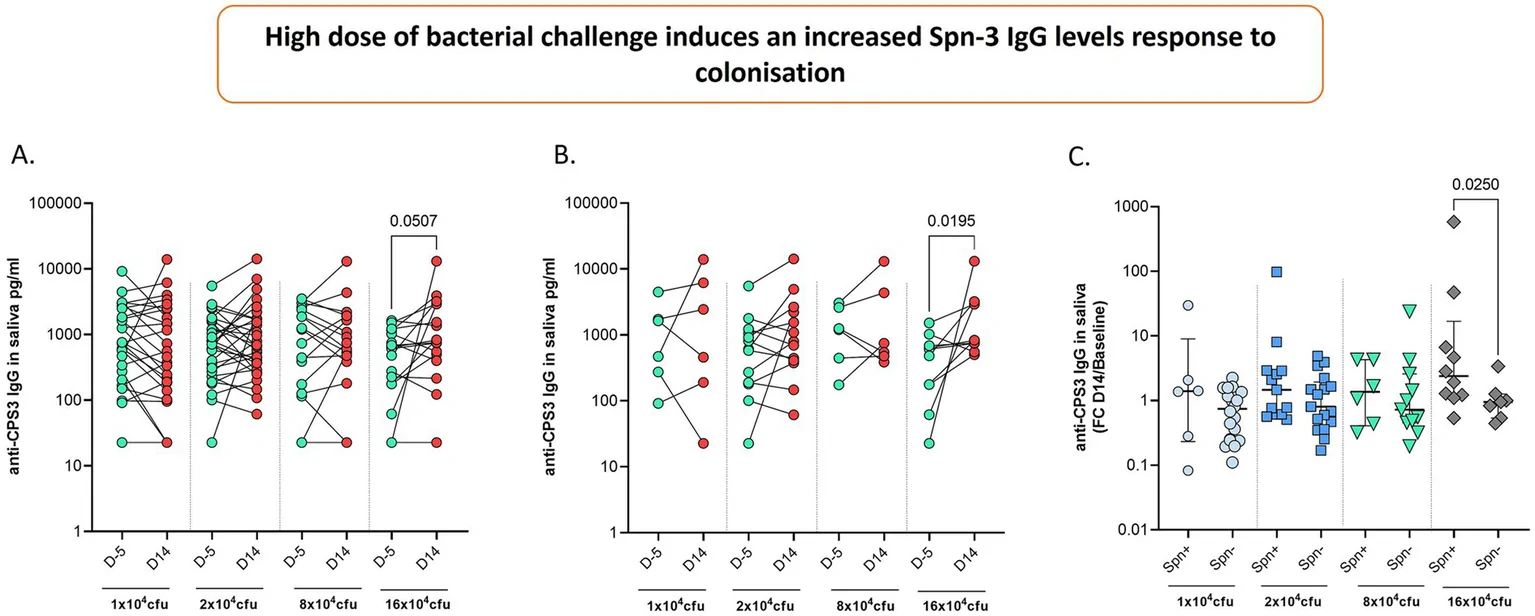
Anti-CPS3 IgG response detected in saliva stratified by the dose-ranging group. **(A)** Anti-CPS3 IgG in saliva at baseline (green) and day 14 (red) for colonised and non-colonised, stratified by dose. Comparisons were performed using the Wilcoxon test. **(B)** Anti-CPS3 IgG in saliva at baseline (green) and day 14 (red) for colonised and non-colonised reporting symptoms, stratified by dose. Comparisons were performed using the Wilcoxon test. **(C)** Fold change in anti-CPS3 IgG from baseline to day 14 in saliva of colonised and non-colonised participants, at 10,000 CFU/naris (circles), 20,000 CFU/naris (squares), 80,000 CFU/naris (diamonds) and 160,000 CFU/naris (triangles). Comparisons were performed using the Mann–Whitney test.

Challenge dose was not associated with a change in saliva anti-CPS3 IgG levels at day 14 from baseline (10,000 CFU/naris, *p* = 0.691; 20,000 CFU/naris, *p* = 0.542; 80,000 CFU/naris *p* = 0.860; 160,000 CFU/naris, *p* = 0.051; Figure 5A). However, when only colonised participants were considered, a significant IgG increase from baseline was observed at 160,000 CFU/naris [GMC IgG at baseline = 338.5 pg./mL (GMC 95% CI 142.8–802.3) vs. GMC at day 14 IgG = 1,282 pg./mL (GMC 95% CI 622.8–2639.0), *p* = 0.019] but not at lower doses (all *p* > 0.05; Figure 5B). A median increase in IgG FC at day 14 from baseline was only observed in the saliva of colonised vs. non-colonised participants inoculated with a dose of 160,000 CFU/naris [median IgG FC = 2.380 (IQR 1.188–16.790) vs. median IgG FC = 0.536 (IQR 0.953–1.261), *p* = 0.025; Figure 5C].

### Colonisation with Spn3 clade Iα but not the other strains showed a trend toward increased saliva IgG immune response

**Figure 6:**
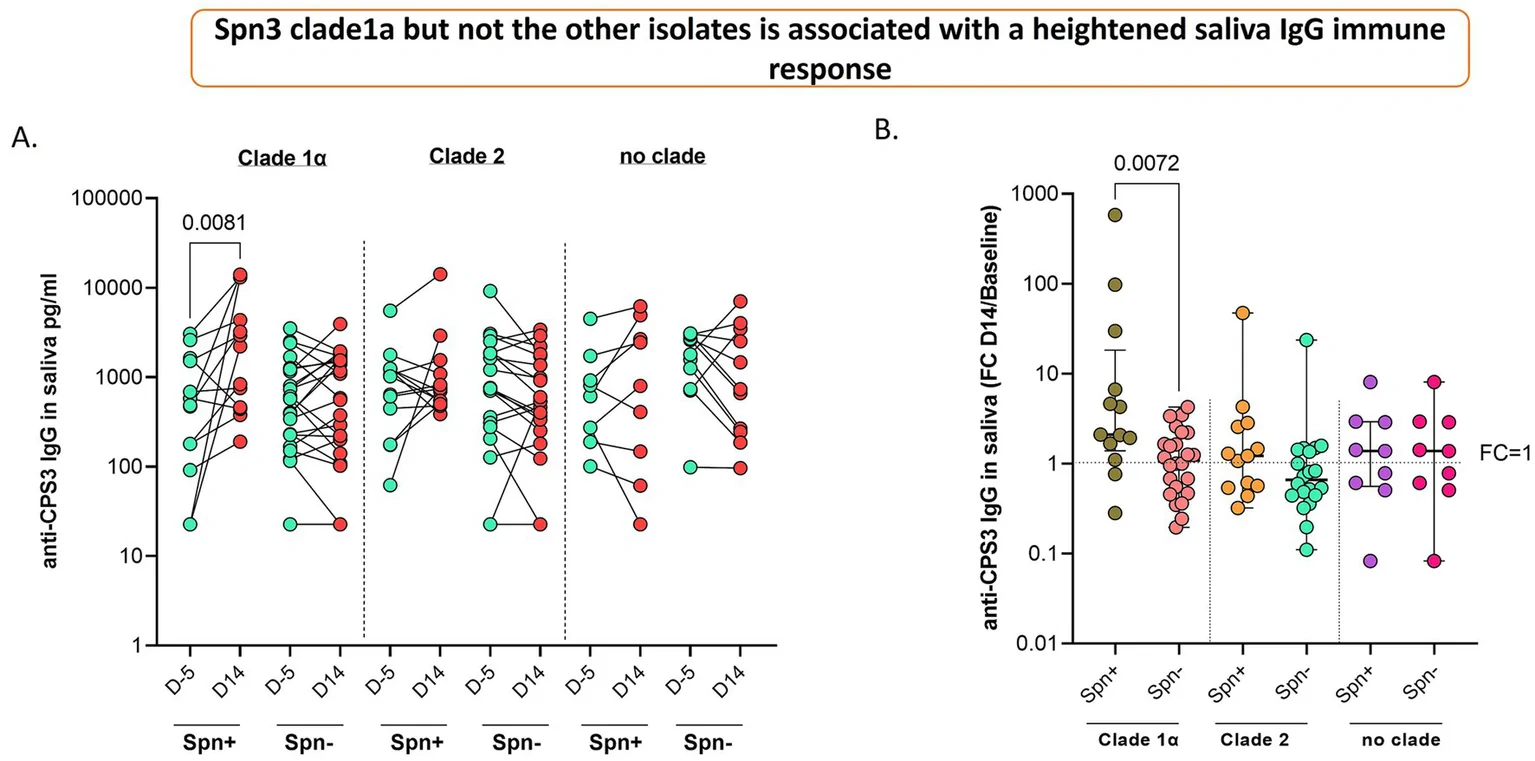
Anti-CPS3 IgG response in saliva for participants stratified by challenge strain. **(A)** Anti-CPS3 IgG (pg/mL) in colonised and non-colonised participants at baseline (green) and day 14 (red) stratified by challenge strain. Comparisons were performed using the Wilcoxon test. **(B)** Fold change in anti-CPS3 IgG from baseline to day 14 in saliva of colonised and non-colonised participants, stratified by challenge strain. Comparisons were performed using the Mann–Whitney test. FC D14/baseline = fold change between day 14 post-challenge and baseline levels.

When stratified by colonisation status and challenge strain (clade Iα Spn3 positive *n* = 13, no clade *n* = 9, clade II *n* = 13), there was no difference in baseline GMC IgG anti-CPS3 between colonised and non-colonised participants in each clade. An increase in GMC IgG responses at day 14 from baseline was observed in participants colonised with Spn3 clade Iα [clade Iα GMC IgG at baseline = 413.4 pg./mL (GMC 95% CI 163.2–1047.0), GMC IgG at day 14 = 1566.0 pg./mL (GMC 95% CI 655.9–3740.0), *p* = 0.008; Figure 6A] but not the other strains. When we compared IgG FC at day 14 from baseline in colonised and non-colonised participants, only Spn3 clade Iα induced an increased IgG response in colonised [median FC, 2.101 (IQR 1.384–18.2) vs. 1.085 (IQR 0.489–2.073), *p* = 0.007] compared to non-colonised participants (Figure 6B).

## Discussion

We have previously shown that the human Spn3 challenge model is feasible and safe when appropriate precautions are in place (Robinson et al., 2022). Establishing pneumococcus within the nasopharynx, a state known as colonisation, depends on its commensal relationship with the human host (Goldblatt et al., 2005). However, in humans, little is known about differences in colonisation between the diverse pneumococcal serotypes, and data on differences between strains of the same serotype are even more scarce. An important question is whether Spn3 has a different colonisation niche and colonisation dynamics from other prevalent serotypes, which could potentially explain why this serotype seems biologically different to others. We have observed in our human challenge model that Spn3 colonisation caused a substantial increase in participants reporting pharyngitis, which has not been observed with our Spn6B, Spn23F or Spn15B challenged participants (over 2000 challenged participants to date) (Robinson et al., 2023). Here, we show that Spn3 can be cultured in saliva following challenge and that saliva and NW are comparable for the detection of Spn3 colonisation (which differs from our previous 6B data), when culture and molecular methods combined are used for bacterial detection. We also report that following challenge, Spn3 leads to higher density in the oropharynx (saliva samples) than that observed with Spn6B.

Similar Spn3 carriage prevalences in saliva and nasal wash samples from experimentally colonised participants suggest that the bacteria can migrate from the nasopharynx to the oropharynx or that contamination of the oropharynx with nasal secretions occurred. We have previously shown that early identification of pneumococcal DNA in saliva (from 1 h) post-challenge is associated with lower rates of subsequent nasal colonisation, determined by nasal washes (Nikolaou et al., 2021). In this study, we observed that Spn3 was detected more frequently in saliva than in nasal washes on day 2 among participants, but this was not more evident in those with symptoms. Using our experimental human challenge model and a Spn6B strain, we have shown that this process occurs within the first 48 h of exposure to pneumococcus (Nikolaou et al., 2021). Our data suggest that for the majority of participants, Spn3 is likely to first establish colonisation in the nasopharynx before migrating to the oropharynx by day 7. The detection of culturable experimental Spn3 post-challenge in saliva, although infrequent, indicates the presence of viable bacteria rather than fragments of dead bacteria transferred from the nasopharynx.

Spn3 density in saliva on day 2 was significantly higher than the salivary density previously observed following experimental Spn6B challenge. This may reflect serotype-specific differences including in colonisation biology (i.e., the oropharynx is a preferable niche for Spn3), mucosal persistence (i.e., the thicker polysaccharide capsule of Spn3 enhances survival in mucus-rich environments) or clearance dynamics (i.e., Spn3 escapes mucosal clearance mechanisms more effectively than Spn6B, allowing accumulation to higher densities). While interpretation is limited due to small participant numbers at a comparative inoculum dose, this high density could lead to local inflammatory responses (Weight et al., 2019) and may be a common part of the causal chain leading to pharyngitis following Spn3 challenge. Our data showed that density was not a sufficient cause of upper respiratory symptoms. Specifically, symptomatic participants following Spn3 colonisation had similar bacterial densities recovered in their saliva and comparable colonisation dynamics to those observed among asymptomatic persons.

Previous studies have reported lower measured capsular polysaccharide antibody levels to Spn3 following PCV administration in both the serum and bronchoalveolar lavage compared to other vaccine serotypes (McLaughlin et al., 2019; Fuji et al., 2024). Consistent with this, following natural infection, salivary IgG levels to Spn3 colonisation were 10–100 times lower than IgG levels to Spn6B colonisation in our previous studies (Pennington et al., 2016). While this could be due to differences in sample types (saliva vs. nasal washes), it could reflect lower immunogenicity of the Spn3 serotype during a colonisation episode or Spn3 capsular shedding overwhelming the immune response (Choi et al., 2016). Moreover, we have shown that in the initial 14 days following colonisation, the host response observed to Spn3 exhibited a threshold immune response, with similar responses at the three lower doses and a heightened response at the 160,000 CFU/naris dose (Robinson et al., 2022). It is unclear from our data whether IgG levels would equilibrate for all four doses at longer follow-up periods or whether the 160,000 CFU/naris represents a higher than physiological inoculum dose for Spn3 and thus has little population-based implications.

While observed in small participant numbers, there was a difference in the salivary anti-CPS IgG antibody response to the clade Iα strain, which was not observed in the ‘no clade’ and clade II strain groups. Genomic studies have shown that the clade II strain has increased in some locations over the past two decades, seemingly unrelated to the use or timing of introduction of Spn3-containing PCV13 (Azarian et al., 2018). The reasons for this are not understood; however, it has been postulated that this may relate to antibiotic resistance profiles (Azarian et al., 2018) or differing antigen profiles (Groves et al., 2019). Murine studies have observed higher density and longer duration colonisation with less virulence in clade II strains (Sekulovic et al., 2024), potentially leading to greater epidemiological success. The absence of a proportional antibody response despite relatively high bacterial density among clade II strains in our study results may reflect clade-specific differences in immune evasion, inflammatory signalling, or antigen presentation compared with clade Iα. Our data suggest that there also may be a differential host immune response between the two clades, with clade II generating a reduced immunological response to colonisation, whilst clade Iα induced stronger immune responses that could lead to quicker colonisation clearance. Of note, our study did not follow participants beyond day 14 and may not have detected the peak of antibody responses, which is expected to occur later. Future studies will investigate whether clade differences exist in carriage duration or ability of PCV13 to prevent colonisation.

There are several limitations to this study. Experimental colonisation involves the direct challenge of pneumococcus into the nose and therefore may not precisely mimic the natural colonisation acquisition process. In this study, saliva samples were used to assess oropharyngeal colonisation; the addition of concurrent bacterial throat swabs would further support these data but were not available for analysis. In addition, it is unclear whether the inoculum dose range utilised in this model was representative of the bacterial challenge occurring during natural colonisation. The bacterial exposure encountered during natural pneumococcal transmission remains poorly researched. Therefore, experimentally administered inoculation doses, although biologically relevant, are best interpreted as controlled colonisation thresholds rather than direct physiological correlates of natural exposure. In terms of the immunological analyses, we did not measure mucosal IgA responses. Consequently, our immunological analyses are restricted to anti-capsular IgG kinetics, and we cannot exclude contributions from IgA-mediated immune mechanisms, including immune exclusion, agglutination, or the activity of IgA2, which is resistant to the pneumococcal IgA1 protease. In addition, IgA responses directed against pneumococcal protein antigens may also contribute to protection against colonisation. Therefore, while anti-capsular IgG responses were not associated with protection from serotype 3 colonisation in this model, the potential contribution of mucosal IgA and other local immune mechanisms remains to be determined in future studies.

Further limitations include the lack of paired serum and saliva samples and the short duration of follow-up sampling post-challenge. Interpretation of subgroup findings should be undertaken cautiously due to the small number of participants colonised in each clade group, as this may have limited detailed immunological differences and affected generalisability. The unavailability of saliva samples at similar time points in previous 6B challenge studies did not allow for comparison of density in the nose (nasal washes) vs. oropharynx (saliva and throat swabs) between these two strains. These studies are ongoing and will allow us to investigate whether the difference in Spn3 biology, already described by others, leads to preferential oropharyngeal colonisation as a niche. This would explain the reported clinical symptoms (sore throat) in this challenge model but not the models with other serotypes.

Despite these limitations, this model successfully allows the detailed examination of pneumococcal colonisation and the local immune response from the point of establishment to termination in both the oropharynx and nasopharynx, which is not fully observable in natural carriage.

## Conclusion

In summary, our findings provide a biological basis for the ongoing circulation of serotype 3 and its subsequent role as the leading cause of adult pneumococcal disease in many settings. Our data also provide a basis for the recent rise of clade II in some locations, particularly in the UK. Future studies should aim to investigate the role of the oropharynx in pneumococcal transmission, particularly for vaccine-escape serotypes such as Spn3. Furthermore, these initial data could help guide the development and testing of future Spn3 vaccines.

## Data Availability

The original contributions presented in the study are included in the article/supplementary material, further inquiries can be directed to the corresponding author.

## Supplementary material

SUPPLEMENTARY FIGURE S1 Corelation between Spn3 density at days 2, 7 and 14 with Spn3 anti-CPS IgG at day 14 post-challenge. Scatter plot demonstrating the correlation between anti-CPS3 levels (pg/mL) and density in saliva at **(A)** day 2, **(B)** day 7 and **(C)** day 14 post challenge. Reported DNA copies/mL are log transformed.

## References

[ref1] Adler H. German E. L. Mitsi E. Nikolaou E. Pojar S. Hales C. . (2021). Experimental human pneumococcal colonisation in older adults is feasible and safe, not immunogenic. Am. J. Respir. Crit. Care Med. 203, 604–613. doi: 10.1164/rccm.202004-1483OC 32941735 PMC7924584

[ref2] Adler H. Nikolaou E. Gould K. Hinds J. Collins A. M. Connor V. . (2019). Pneumococcal colonization in healthy adult research participants in the conjugate vaccine era, United Kingdom, 2010–2017. J. Infect. Dis. 219, 1989–1993. doi: 10.1093/infdis/jiz034, 30690468 PMC6534187

[ref3] Arguedas A. Trzciński K. O’Brien K. L. Ferreira D. M. Wyllie A. L. Weinberger D. . (2020). Upper respiratory tract colonization with *Streptococcus pneumoniae* in adults. Expert Rev. Vaccines 19, 353–366. doi: 10.1080/14760584.2020.1750378, 32237926

[ref4] Azarian T. Mitchell P. K. Georgieva M. Thompson C. M. Ghouila A. Pollard A. J. . (2018). Global emergence and population dynamics of divergent serotype 3 CC180 pneumococci. PLoS Pathog. 14:e1007438. doi: 10.1371/journal.ppat.1007438, 30475919 PMC6283594

[ref5] Azzari C. Moriondo M. Indolfi G. Cortimiglia M. Canessa C. Becciolini L. . (2010). Realtime PCR is more sensitive than multiplex PCR for diagnosis and serotyping in children with culture negative pneumococcal invasive disease. PLoS One 5:e9282. doi: 10.1371/journal.pone.0009282, 20174571 PMC2824814

[ref6] Bogaert D. de Groot R. Hermans P. (2004). *Streptococcus pneumoniae c*olonisation: the key to pneumococcal disease. Lancet Infect. Dis. 4, 144–154. doi: 10.1016/S1473-3099(04)00938-7, 14998500

[ref7] Bomar L. Brugger S. D. Lemon K. P. (2018). Bacterial microbiota of the nasal passages across the span of human life. Curr. Opin. Microbiol. 41, 8–14. doi: 10.1016/j.mib.2017.10.023, 29156371 PMC5862745

[ref8] Carvalho M. d. G. S. Tondella M. L. McCaustland K. Weidlich L. McGee L. Mayer L. W. . (2007). Evaluation and improvement of real-time PCR assays targeting *lytA*, *ply*, and *psaA* genes for detection of pneumococcal DNA. J. Clin. Microbiol. 45, 2460–2466. doi: 10.1128/JCM.02498-06, 17537936 PMC1951257

[ref9] Chaguza C. Senghore M. Bojang E. Lo S. W. Ebruke C. Gladstone R. A. . (2021). Carriage dynamics of pneumococcal serotypes in naturally colonized infants in a rural African setting during the first year of life. Front. Pediatr. 8:898. doi: 10.3389/fped.2020.587730, 33489998 PMC7820366

[ref10] Choi E. H. Zhang F. Lu Y.-J. Malley R. (2016). Capsular polysaccharide (CPS) release by serotype 3 pneumococcal strains reduces the protective effect of anti-type 3 CPS antibodies. Clin. Vaccine Immunol. 23, 162–167. doi: 10.1128/CVI.00591-15, 26677201 PMC4744920

[ref11] Cundell D. R. Weiser J. N. Shen J. Young A. Tuomanen E. I. (1995). Relationship between colonial morphology and adherence of *Streptococcus pneumoniae*. Infect. Immun. 63, 757–761. doi: 10.1128/iai.63.3.757-761.1995, 7868244 PMC173067

[ref12] Ferreira D. M. Neill D. R. Bangert M. Gritzfeld J. F. Green N. Wright A. K. A. . (2013). Controlled human infection and rechallenge with *Streptococcus pneumoniae* reveals the protective efficacy of carriage in healthy adults. Am. J. Respir. Crit. Care Med. 187, 855–864. doi: 10.1164/rccm.201212-2277OC, 23370916 PMC3707375

[ref13] Fuji N. Pham M. Kaur R. Pichichero M. E. (2024). Serotype 3 antibody response and antibody functionality comapred to serotype 19A following 13-valent pneumococcal conjugate immunization in children. Pediatr. Infect. Dis. J. 43, 294–300. doi: 10.1097/INF.0000000000004192 38048644 PMC10922043

[ref14] Gessner B. D. Jiang Q. van Werkhoven C. H. Sings H. L. Webber C. Scott D. . (2019). A post-hoc analysis of serotype-specific vaccine efficacy of 13-valent pneumococcal conjugate vaccine against clinical community acquired pneumonia from a randomized clinical trial in the Netherlands. Vaccine 37, 4147–4154. doi: 10.1016/j.vaccine.2019.05.065, 31155413

[ref15] Gladstone R. Jefferies J. Faust S. Clarke S. (2012). Sampling methods for the study of pneumococcal carriage: a systematic review. Vaccine 30, 6738–6744. doi: 10.1016/j.vaccine.2012.08.080, 22981760

[ref16] Goldblatt D. Hussain M. Andrews N. Ashton L. Virta C. Melegaro A. . (2005). Antibody responses to nasopharyngeal carriage of *Streptococcus pneumoniae* in adults: a longitudinal household study. J. Infect. Dis. 192, 387–393. doi: 10.1086/431524, 15995951

[ref17] Goldblatt D. Plikaytis B. D. Akkoyunlu M. Antonello J. Ashton L. Blake M. . (2011). Establishment of a new human pneumococcal standard reference serum, 007sp. Clin. Vaccine Immunol. 18, 1728–1736. doi: 10.1128/CVI.05252-11, 21852547 PMC3187044

[ref18] Groves N. Sheppard C. L. Litt D. Rose S. Silva A. Njoku N. . (2019). Evolution of *Streptococcus pneumoniae* serotype 3 in England and Wales: a major vaccine evader. Genes. 10:845. doi: 10.3390/genes10110845, 31731573 PMC6896183

[ref19] Levine H. Zarka S. Dagan R. Sela T. Rozhavski V. Cohen D. I. . (2012). Transmission of *Streptococcus pneumoniae* in adults may occur through saliva. Epidemiol. Infect. 140, 561–565. doi: 10.1017/S0950268811000884, 21676361

[ref20] Lieberman D. Shleyfer E. Castel H. Terry A. Harman-Boehm I. Delgado J. . (2006). Nasopharyngeal versus oropharyngeal sampling for isolation of potential respiratory pathogens in adults. J. Clin. Microbiol. 44, 525–528. doi: 10.1128/JCM.44.2.525-528.2006, 16455908 PMC1392694

[ref21] Løchen A. Croucher N. J. Anderson R. M. (2020). Divergent serotype replacement trends and increasing diversity in pneumococcal disease in high income settings reduce the benefit of expanding vaccine valency. Sci. Rep. 10:18977. doi: 10.1038/s41598-020-75691-5, 33149149 PMC7643077

[ref22] Luck J. N. Tettelin H. Orihuela C. J. (2020). Sugar-coated killer: serotype 3 pneumococcal disease. Front. Cell. Infection Micorbiol. 10:613287. doi: 10.3389/fcimb.2020.613287 PMC7786310 33425786

[ref23] McLaughlin J. M. Jiang Q. Gessner B. D. Swerdlow D. L. Sings H. L. Isturiz R. E. . (2019). Pneumococcal conjugate vaccine against serotype 3 pneumococcal pneumonia in adults: a systematic review and pooled analysis. Vaccine 37, 6310–6316. doi: 10.1016/j.vaccine.2019.08.059, 31522807

[ref24] Murad C. Dunne E. M. Sudigdoadi S. Fadlyana E. Tarigan R. Pell C. L. . (2019). Pneumococcal carriage, density, and co-colonization dynamics: a longitudinal study in Indonesian infants. Int. J. Infect. Dis. 86, 73–81. doi: 10.1016/j.ijid.2019.06.024, 31247341

[ref25] Nikolaou E. German E. L. Blizard A. Howard A. Hitchins L. Chen T. . (2021). The nose is the best niche for detection of experimental pneumococcal colonisation in adults of all ages, using nasal wash. Sci. Rep. 11:18279. doi: 10.1038/s41598-021-97807-1, 34521967 PMC8440778

[ref26] Nikolaou E. Jochems S. P. Mitsi E. Pojar S. Blizard A. Reiné J. . (2021). Experimental human challenge defines distinct pneumococcal kinetic profiles and mucosal responses between colonized and non-colonized adults. MBio 12:e02020. doi: 10.1128/mBio.02020-20, 33436429 PMC7844534

[ref27] Pennington S. H. Pojar S. Mitsi E. Gritzfeld J. F. Nikolaou E. Solórzano C. . (2016). Polysaccharide-specific memory B cells predict protection against experimental human pneumococcal carriage. Am. J. Respir. Crit. Care Med. 194, 1523–1531. doi: 10.1164/rccm.201512-2467OC, 27403678 PMC5215029

[ref28] Pick H. Daniel P. Rodrigo C. Bewick T. Ashton D. Lawrence H. . (2020). Pneumococcal serotype trends, surveillance and risk factors in UK adult pneumonia, 2013–18. Thorax 75, 38–49. doi: 10.1136/thoraxjnl-2019-213725, 31594801

[ref29] Robinson R. E. Mitsi E. Nikolaou E. Pojar S. Chen T. Reiné J. . (2022). Human infection challenge with serotype 3 pneumococcus. Am. J. Respir. Crit. Care Med. 206, 1379–1392. doi: 10.1164/rccm.202112-2700OC, 35802840

[ref30] Robinson R. E. Myerscough C. He N. Hill H. Shepherd W. A. Gonzalez-Dias P. . (2023). Comprehensive review of safety in experimental human pneumococcal challenge. PLoS One 18:e0284399. doi: 10.1371/journal.pone.0284399, 37141259 PMC10159102

[ref31] Rylance J. de Steenhuijsen Piters W. A. Mina M. J. Bogaert D. French N. Ferreira D. M. . (2019). Two randomized trials of the effect of live attenuated influenza vaccine on pneumococcal colonization. Am. J. Respir. Crit. Care Med. 199, 1160–1163. doi: 10.1164/rccm.201811-2081LE, 30758980 PMC6515882

[ref32] Sekulovic O. Gallagher C. Lee J. Hao L. Zinonos S. Tan C. Y. . (2024). Evidence of reduced virulence and increased colonization among pneumococcal strains of serotype 3 clade II lineage in mice. J. Infect. Dis.:jiae038. doi: 10.1093/infdis/jiae038 PMC11272092 39052735

[ref33] Sings H. L. de Wals P. Gessner B. D. Isturiz R. Laferriere C. McLaughlin J. M. . (2019). Effectiveness of 13-valent pneumococcal conjugate vaccine against invasive disease caused by serotype 3 in children: a systematic review and meta-analysis of observational studies. Clin. Infect. Dis. 68, 2135–2143. doi: 10.1093/cid/ciy920, 30357326 PMC6541704

[ref34] Trzciński K. Bogaert D. Wyllie A. Chu M. L. J. N. van der Ende A. Bruin J. P. . (2013). Superiority of trans-oral over trans-nasal sampling in detecting *Streptococcus pneumoniae* colonization in adults. PLoS One 8:e60520. doi: 10.1371/journal.pone.0060520, 23555985 PMC3610877

[ref35] Warren J. L. Weinberger D. M. (2020). Estimating serotype-specific efficacy of pneumococcal conjugate vaccines using hierarchical models. Epidemiology 31, 259–262. doi: 10.1097/EDE.0000000000001135, 31913908 PMC7508209

[ref36] Watt J. P. O'Brien K. L. Katz S. Bronsdon M. A. Elliott J. Dallas J. . (2004). Nasopharyngeal versus oropharyngeal sampling for detection of pneumococcal carriage in adults. J. Clin. Microbiol. 42, 4974–4976. doi: 10.1128/JCM.42.11.4974-4976.2004, 15528682 PMC525247

[ref37] Weight C. M. Venturini C. Pojar S. Jochems S. P. Reiné J. Nikolaou E. . (2019). Microinvasion by *Streptococcus pneumoniae* induces epithelial innate immunity during colonisation at the human mucosal surface. Nat. Commun. 10:3060. doi: 10.1038/s41467-019-11005-2, 31311921 PMC6635362

[ref38] Weiser J. N. Ferreira D. M. Paton J. C. (2018). *Streptococcus pneumoniae*: transmission, colonization and invasion. Nat. Rev. Microbiol. 16, 355–367. doi: 10.1038/s41579-018-0001-8, 29599457 PMC5949087

